# A Generalizable Tool for Predicting Developmental Phenology for Wild Ecotherms

**DOI:** 10.1002/ece3.74142

**Published:** 2026-08-04

**Authors:** Morgan M. Sparks, Brian C. Leavell, Bryan M. Maitland

**Affiliations:** ^1^ Boise Aquatic Sciences Laboratory, Rocky Mountain Research Station USDA Forest Service Boise Idaho USA; ^2^ Smithsonian Tropical Research Institute Panama City Panama; ^3^ Department of Biological Sciences Purdue University West Lafayette Indiana USA

**Keywords:** developmental phenology, hatchR, local adaptation, R, reaction norm, Shiny

## Abstract

Development in poikilothermic organisms is strongly temperature‐dependent, particularly during early life stages prior to exogenous feeding. This thermally constrained physiology produces a near‐mechanistic relationship between ambient temperature and developmental rate, allowing for predictive models of phenology. However, traditional models—often developed under constant laboratory temperatures—struggle to account for the variable thermal regimes experienced by wild populations. Effective value models were created to address this issue and are a framework that integrates diel temperature data to predict developmental timing under fluctuating conditions. Building on this foundation, the R package **hatchR** was developed for fishes to operationalize the effective value model and enable its widespread application. In this paper, we expand the scope of **hatchR**, demonstrating its applicability across a broad range of poikilothermic taxa including amphibians, reptiles, insects, crustaceans, copepods, and starfishes. We also illustrate how this framework supports both applied management goals—such as predicting hatching windows for conservation and monitoring—and basic research on ecological and evolutionary questions including phenological plasticity, thermal adaptation, and ecological interactions. By providing a generalizable, open‐source tool, **hatchR** bridges the gap between physiological theory and real‐world application, offering a robust platform for predicting developmental phenology in diverse and changing environments.

## Graphical abstract

Custom hatch timing models developed for four North American *Lithobates* frog species. Models are parameterized from Moore (1939). Panels (A, B) shows differential developmental rates under varying thermal regimes and G × E interactions.

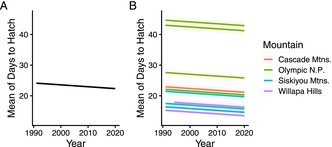

## 1 Introduction

Because poikilothermic organisms rely on external sources to regulate their body temperature, the physiological processes governing their growth and development are tightly coupled to ambient thermal conditions. This relationship is especially strong during early life stages prior to exogenous feeding and is modulated by genetic and maternal effects (West‐Eberhard 2003). Although the absolute rate of development can vary widely among species and populations, most poikilotherms exhibit a consistent and often non‐linear thermal performance curve that describes development as a function of temperature (Schoolfield et al. 1981; Angilletta Jr. 2006). Within an organism's typical thermal optima, developmental rates can be effectively approximated with power law or exponential models that capture accelerating developmental rates with rising temperature.

Historically, models of developmental phenology in wild populations have relied on the accumulation of degree days (thermal sums) above a species‐specific threshold. While widely used, degree‐day models are fundamentally linear and fail to account for the non‐linear nature of development, leading to biased predictions under fluctuating temperature regimes (Neuheimer and Taggart 2007; Steel et al. 2012). This is particularly problematic for long‐developing species or for organisms in variable climates, where embryos incubating at colder temperatures often require fewer thermal units to complete development than those incubating in warmer environments (Quinn 2018). Finally, developmental reaction norms may vary across populations due to local adaptation or phenotypic plasticity, further complicating predictions (Angilletta Jr. 2009; Sparks et al. 2017).

To address these limitations in fishes, Sparks et al. (2019) introduced the effective value model—an approach that captures the non‐linear, temperature‐dependent nature of development by integrating experimental data into a diel developmental rate function. Building on that work, the R package **hatchR** (Sparks et al. 2026) was developed to operationalize the effective value framework and make it accessible to researchers and managers. **hatchR** uses temperature‐performance curves generated from lab‐based thermal rearing trials to estimate the proportion of development achieved each day under observed temperature conditions. These diel contributions are summed until a cumulative threshold is reached, marking the completion of a developmental stage. The package includes both an R‐based workflow and a user‐friendly Shiny web interface (R Core Team 2024; Sparks et al. 2025) to facilitate broad adoption across research and management communities (https://bmait101.github.io/hatchR/index.html).

While **hatchR** was originally developed for coldwater fishes, the approach is broadly applicable to any poikilothermic organism whose development conforms to a monotonic or unimodal temperature–rate relationship, including many amphibians, reptiles, and invertebrates. In this manuscript, we expand the taxonomic scope of **hatchR**, compiling thermal developmental data from the literature and demonstrating how effective value models can be applied to diverse taxa. We also present three case studies illustrating how **hatchR** can inform ecological, evolutionary, and management questions—ranging from understanding thermal adaptation to forecasting hatch timing in conservation planning. These applications are increasingly relevant as ecosystems, especially aquatic ones, face intensifying and interacting stressors, including variable climates, habitat alteration, and invasive species, all of which can disrupt developmental timing and population dynamics (Craig et al. 2017; Pinsky et al. 2019).

## 2 Methods

### 2.1 Effective Value Models Overview

Effective value models estimate developmental progress based on empirically derived relationships between temperature and development rate. These relationships are typically parameterized by raising organisms at multiple constant temperatures in laboratory settings, then fitting a non‐linear function that captures the temperature‐dependence of development (see Table 1 for examples). This function can then be reciprocated to produce diel “effective values”—units of development—that accumulate over time (Sparks et al. 2019).

**TABLE 1:** Putative sources for effective value parameterization using fit_model() in **hatchR** for a broad array of taxa. While et al. (2018) and Pritchard and Leggott (1987) are broader reviews with multiple species and are flagged as not yet vetted for full reciprocal model implementation. Users may need to extract or adapt published equations to align with the effective value framework.

Class	Order	Genus	Species	Study
Amphibia	Anura	*Ascaphus*	*A. truei*	Brown (1975)
		*Lithobates*	*L. clamitans, L. palustris, L. pipiens, L. sylvaticus*	Moore (1939)
	Urodela	*Ambystoma*	*A. gracile*	Brown (1976)
Asteroidea Cephalopoda	Valvatida	*Acanthaster*	*A. planci*	Hoegh‐Guldberg and Pearse (1995)
		*Asterina*	*A. miniata*	
		*Odontaster*	*O. meridionalis, O. validus*	
	Myopsida	*Loligo*	*L. reynaudii, L. vulgaris*	Márquez et al. (2021)
	Octopoda	*Octopus*	*O. mimus, O. vulgaris*	
	Oegopsida	*Illex*	*I. coindetii, I. illecebrosus*	
		*Ommastrephes*	*O. bartramii*	
		*Todarodes*	*T. pacificus*	
Copepoda	Calanoida	10 genera	28 species	Forster et al. (2011)
	Cyclopoida	*Limnoithona*	*L. tetraspina*	
		*Oithona*	*O. davisae*	
	Harpacticoida	*Mesochra*	*M. lilljeborgi*	
		*Microsetella*	*M. norvegica*	
	Poecilostomatoida	*Oncaea*	*O. venusta*	
Insecta	Coleoptera	*Colaphellus*	*C. bowringi*	Tang et al. (2017)
	Culicidae	*Aedes*	*A. sticticus, A. vexans*	Developmental equations in Pritchard and Leggott (1987)
		*Anopheles*	*A. quadrimaculatus*	
		*Toxorhynchites*	*T. brevipalpis*	
	Odonata	7 genera	14 species	
	Plecoptera	*Capnia*	*C. atra*	Brittain and Mutch (1984)
		*Capnia*	*C. bifrons*	Elliott (1986)
		*Mesocapnia*	*M. oenone*	Brittain and Mutch (1984)
		*Nemurella*	*N. pictetii*	Brittain (1978), Elliott (1984)
		*Taeniopteryx*	*T. nebulosa*	Brittain (1977)
Malacostraca	Decapoda	*Pontastacus*	*P. leptodactylus*	Aydın and Dilek (2004)
Reptilia	Squamata	*Podarcis*	*P. muralis*	Van Damme et al. (1992)
		*Sceloporus*	*S. undulatus*	Angilletta et al. (2000)
	Testudines	*Mauremys*	*M. reevesii*	Du et al. (2007)
			181 species	141 studies in While et al. (2018)

Each day's developmental contribution is calculated using the general power‐law form:

$$\text{EffectiveValu}e_{i}=1/\exp\left(\mathrm{lo}g_{e}a-\mathrm{lo}g_{e}\left(\text{Temperatur}e_{i}-b\right)\right)$$

where *i* is the index for each day, *a* and *b* are empirically derived parameters, and temperature is the average for that day. Development is complete once the cumulative sum of diel effective values reaches one:

$$\sum_{i=1}^{n}\text{EffectiveValu}e_{i}=1$$

Effective value models are premised on experimental data, often using fixed temperature treatments. However, because they accurately account for the diel contribution to development, they may be applied to fluctuating regimes. Sparks et al. (2017) reparameterized Model 2 from Beacham and Murray (1990) for western Alaskan sockeye salmon ( *Oncorhynchus nerka* ) using common garden data and demonstrated how effective value models can be parameterized using average temperatures over the course of development to predict hatch timing for embryos receiving diel fluctuating temperatures, with near‐perfect concordance between model estimates and empirical data. Finally, while **hatchR** generally operates assuming the user is predicting at the diel time‐step, custom models can be parameterized for other time‐steps, such as hourly, which is especially useful for rapidly developing species.

**FIGURE 1:**
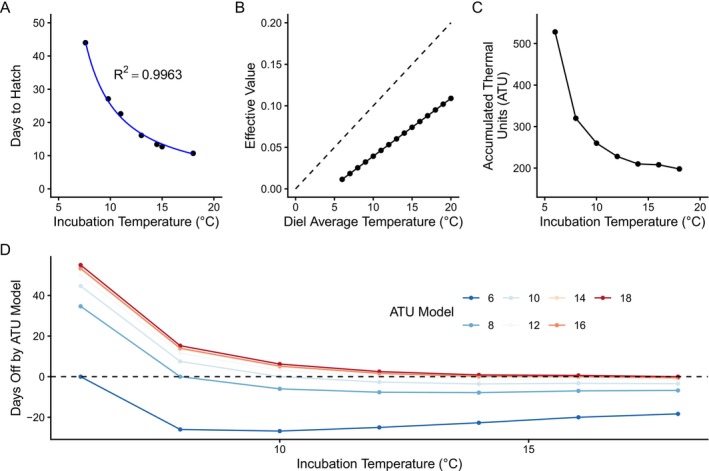
Custom hatching phenology model showing parameterization for coastal tailed frogs ( *Ascaphus truei* ). Panel (A) represents the non‐linear performance curve and raw data used to generate the effective value model. Panel (B) illustrates the effective values for diel temperatures between 6°C and 20°C. A dashed line with a 0.01 increase for every degree increase is included for reference. Panel (C) uses the model fit from Panel (A) to convert to an accumulated thermal unit (ATU) model (number of days to hatch × diel temperature), showing a variable threshold for accumulated units to hatch. Panel (D) shows differences in modeled days to hatch (Panel A) when an accumulated thermal unit model parameterized at a certain temperature is used across the range of temperatures from Panel (A). ATU models make unbiased predictions when the temperature the model is parameterized at (ATU Model in the figure) matches the incubation temperature, but becomes increasingly biased when those numbers become more disparate.

To illustrate how a model can be parameterized for non‐fish organisms, we developed a custom effective value model for the coastal tailed frog ( *Ascaphus truei* ), a cold‐adapted amphibian widespread in forested streams of western North America (Figure 1A,B). We used **hatchR**'s fit_model() function, which fits power‐law models (specifically, Model 2 following Beacham and Murray 1990) to thermal performance data. While **hatchR** defaults to the power‐law framework, users may supply any model formulation via the predict_phenology() function, enabling flexibility for taxa or traits that require alternative temperature‐development functions.

### 2.2 Effective Value Models Versus Threshold Models

In addition to demonstrating how an effective value model might be parameterized, we also use the same underlying data for coastal tailed frogs to show how accumulated thermal models can be highly inaccurate and biased when not used appropriately. Because there is no constant thermal unit threshold at which coastal tailed frogs hatch (Figure 1C)—as is the case with most poikilotherms—when using accumulated thermal unit (ATU) models in wild environments, predictions will only be accurate when the parameterized ATU model (Figure 1C) closely matches the experienced temperature in situ (Figure 1D). These differences are most pronounced when models parameterized at very cold temperatures are used in very warm environments or vice‐versa (> 40 days). However, even models that more closely match the wild environment, for example an accumulated thermal unit model parameterized at 10°C, but used for a thermal regime that is actually 8°C, is off by 7.5 days or 18.75% of the 40 day empirical developmental period.

### 2.3 Validating Models With Empirical Results

To demonstrate how models could be parameterized using average temperatures to accurately predict development with in situ thermal regimes, we used data from Sparks et al. (2017). Briefly, Sparks et al. (2017) reared populations of sockeye salmon from highly divergent thermal environments in a common garden experimental design. Fertilized embryos were transported from their natal environments the day of fertilization and reared in experimental thermal regimes that were chosen to reflect empirical regimes measured in their home locations (Woody Island and Pedro Ponds, Alaska). The regimes represent a highly thermally variable and warmer, wind‐mixed island shore habitat (Woody Island) and cool and stable spring‐fed ponds (Pedro Ponds). The exact details of the treatments are available in Sparks et al. (2017), but the treatments were designed to either match the empirical records of the thermal environment measured (WI_AVG, WI_COLD, PP_AVG) at those locations or were modeled to reflect warming temperatures over the next century (WI_MIROC, PP_MIROC).

**FIGURE 2:**
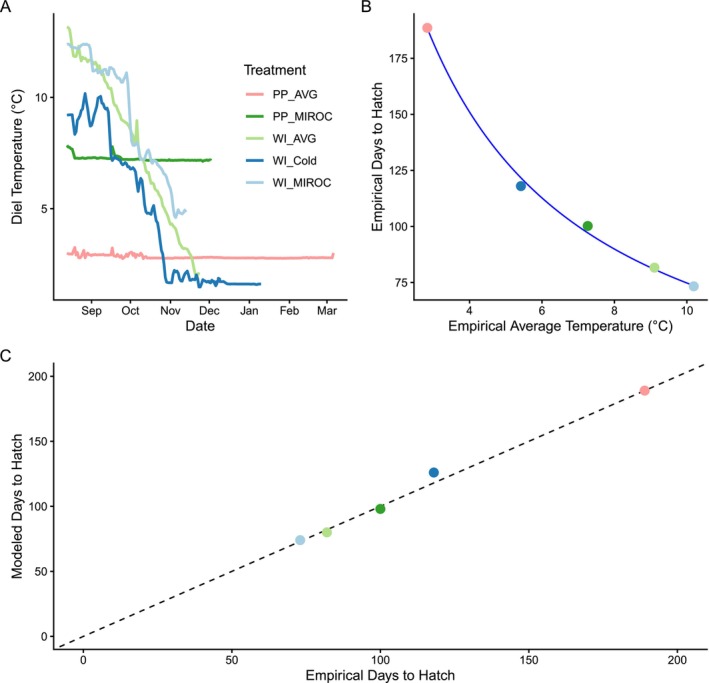
Model parameterization and prediction for variable thermal regimes used in Sparks et al. (2017). Panel (A) shows empirical thermal regimes used to rear sockeye salmon ( *Oncorhynchus nerka* ) with regimes of different averages and variance (regimes end when all fish were hatched). Panel (B) shows a developmental model parameterized using the average temperatures from those regimes. Panel (C) shows the days to hatch predicted with the custom model and diel temperatures from the empirical thermal regimes and compares them to empirical average days to hatch from the experiment. The dashed line shows a 1:1 relationship.

We used the thermal regimes from the common garden experiment (Figure 2A) and summarized average days to hatch and the corresponding average temperature in the experiment for the populations in the study. Using those empirical measures, we then parameterized a custom model using the fit_model() function (Figure 2B) and predicted days to hatch using the diel temperatures recorded in the experiment, which we then compared with empirical results (Figure 2C). There was high correlation between the empirical and predicted days to hatch (*r* = 0.996) and the average absolute difference between modeled and empirical results was small (2.6 days), suggesting model performance was highly concordant with empirical observations. The scenario where the model performed worst was WI_Cold, the most variable of the scenarios (mean = 5.418, SD = 3.136), and predicted a mean hatch date 8 days later (6.8%) than observed.

### 2.4 Using hatchR to Predict Phenology

**hatchR** requires two core inputs: a vector of diel average temperatures and a corresponding vector of calendar dates. These data are typically collected from field‐deployed temperature loggers. For loggers that record sub‐diel temperatures, **hatchR** includes the function summarize_temp() to compute diel means. Data completeness and consistency can be checked using plot_check_temp() and check_continuous() to ensure there are no gaps or anomalies in the temperature record.

Developmental phenology can be predicted in two ways using **hatchR**. The predict_phenology() function estimates the date at which development completes, given a known reproductive event (e.g., oviposition or spawning; using the spawn.date argument) and diel temperature data. Alternatively, the predict_spawn() function works in reverse—estimating the likely date of a reproductive event based on a known or observed hatch/emergence date. This is especially useful for retrospective analyses or for estimating breeding windows from field observations. To run models in **hatchR** users need to provide:

1. Some record of temperatures that can be summarized to an average diel time‐step.
2. A date that overlaps with the temperature record for when mating (predict_phenology()) or development (predict_spawn()) occurred.
3. Experimental data to create custom effective value model parameterizations (or, in the case of some fishes, use included models).

## 3 Case Studies

Here we demonstrate how the effective value modeling framework is broadly applicable across poikilothermic taxa and can be used to address a wide array of ecological, evolutionary, and management questions (also, see Sparks et al. (2026) for an example of instream work‐window planning during a sensitive developmental period for a threatened fish species). While this manuscript focuses on three illustrative case studies, many additional opportunities exist. To this end, we also provide a non‐exhaustive compilation of species for which thermal developmental data are available in the peer‐reviewed or gray literature (Table 1). These sources can be used to parameterize custom models via the fit_model() function in **hatchR** (as demonstrated in Figures 1 and 2), allowing users to build tailored effective value models for diverse taxa. The examples span seven taxonomic classes—amphibians, reptiles, insects, crustaceans, copepods, cephalopods, and asteroids—and demonstrate the breadth of systems to which this approach can be applied (for examples with fishes, see Sparks et al. (2026)). While this manuscript focuses on three case studies, the ecological, evolutionary, and management applications are broad—spanning phenological mismatch and climate vulnerability assessments to invasive species risk modeling and conservation timing of sensitive life stages.

### 3.1 Ecological Application: Spatial and Temporal Trends in Development

Examining variation in development across space and time is critical to understand how organisms respond to ecological factors such as variable climates (Pinsky et al. 2019). Here, we demonstrate an application of **hatchR** to predict hatching times from a spatio‐temporally extensive dataset. Using these predictions, we then show how one can examine putative drivers of variation in development and discuss their application in forecasting future responses.

We modeled variation in developmental timing for coastal tailed frogs ( *A. truei* ) across their range in the Pacific Northwest. Using spatially‐ and temporally‐varying oviposition records from Karraker et al. (2006) (N. Karraker, personal communication) and our earlier parameterized effective value model (Figure 1A,B), we modeled diel development across 30 years (1991–2020) using modeled stream temperature data from Siegel et al. (2023).

After making hatch timing predictions, the dataset included 480 observations of predicted days to hatch, drawn from 11 stream reaches (labeled with Common Identifiers, that is, COMIDs from the National Hydrogrpahy Dataset) (McKay et al. 2012) nested within four mountain ranges: Olympic National Park, the Cascade Mountains, the Siskiyou Mountains, and the Willapa Hills. Each stream reach had between 20 and 30 years of predictions.

We evaluated eight candidate linear models that varied in the inclusion and interaction of year, stream reach, and mountain range effects. Based on adjusted *R* ^2^, AICc, and RMSE, the best‐supported model included year and stream reach (COMID) as additive fixed effects: days_to_hatch ~ year + stream reach.

**FIGURE 3:**
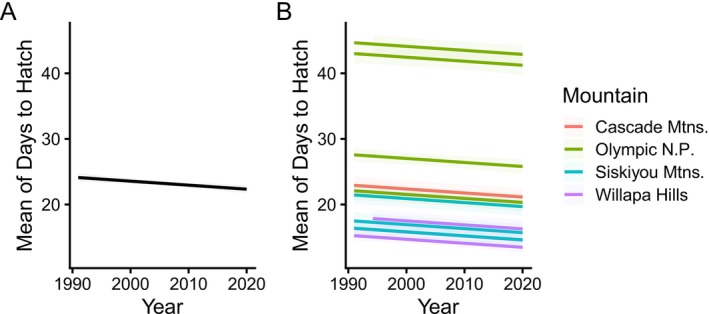
Modeled changes in developmental timing for coastal tailed frogs ( *Ascaphus truei* ) from 1991 to 2020s with stream temperatures from Siegel et al. (2023) using a model parameterized in this study (Figure 1). Panel (A) shows a marginal mean trend across all sites and a significant decline in days to hatching over time. Panel (B) shows site‐ and reach‐specific (COMID) trends in predicted days to hatch, colored by mountain range. Shaded ribbons represent 95% confidence intervals.

This model explained ~87% of the variation in developmental timing (adjusted *R* ^2^ = 0.866), with year having a significant weak and negative effect (−0.06 days/year, *p* = 0.001), indicating an overall trend toward faster development consistent with regional warming (Figure 3A). Stream reach effects captured the persistent differences in thermal regimes among streams, with estimated marginal means ranging from ~15 to ~44 days to hatch across sites (Figure 3B).

Our results indicate developmental rates were highly variable over different physical habitats (streams originating in lowland hills compared with those originating in mountain ranges with permanent snow fields), with sites in Olympic National Park consistently having the longest development times and the greatest interannual variability (SD ~11 days), while lowland streams in the Willapa Hills developed fastest with minimal variability (SD < 3 days). These results demonstrate the capacity of **hatchR** to detect spatiotemporal phenological variation and forecast potential shifts under shifting climate patterns.

### 3.2 Phylogenetic Application: Species Differences in Developmental Rates

Closely related species often diverge in their thermal tolerances and developmental strategies as part of niche differentiation (Angilletta Jr. 2009). These differences can be visualized through reaction norms that capture genotype‐by‐environment (G × E) interactions, often derived from common garden or reciprocal transplant experiments. While such experiments are ideal for isolating genetic and plastic components of trait variation, they are logistically demanding and rarely available for many taxa.

**hatchR** offers a complementary approach by generating species‐specific developmental models from published thermal rearing studies and applying them to shared temperature regimes. While this does not replace controlled experiments, it enables estimation of thermal reaction norms for development across species, even when the original experiments were conducted independently. By visualizing differences in effective value slopes and phenological outcomes under shared conditions, **hatchR** can highlight how developmental traits vary with phylogeny and ecological strategy.

To demonstrate this approach, we leveraged data from Moore (1939) to parameterize developmental models for four widely distributed North American frog species in the genus *Lithobates*: wood frog ( *L. sylvaticus* ), pickerel frog ( *L. palustris* ), northern leopard frog ( *L. pipiens* ), and green frog ( *L. clamitans* ). These species co‐occur in parts of eastern North America but differ in their breeding phenology, habitat associations, and thermal niches. For example, wood frogs are early breeders in cold, ephemeral wetlands, while green frogs breed later in more permanent, warmer waters (Moore 1939).

**FIGURE 4:**
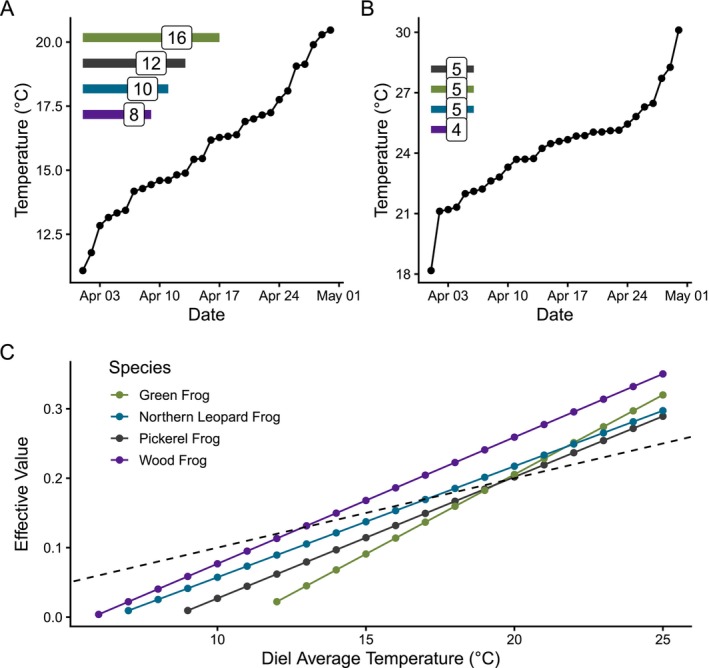
Custom hatch timing models developed for four North American *Lithobates* frog species. Models are parameterized from Moore (1939) and phenology is predicted using two synthetic temperature variable regimes: Panel (A) shows predictions under a cooler regime (mean = 16°C), while Panel (B) shows predictions under a warmer regime (mean = 24°C). Horizontal bars and numbers indicate the predicted days to hatch for a given thermal regime, while the dotted line represents the diel mean temperature. Panel (C) displays the effective value curves for each species—linearized thermal reaction norms. The crossover in rank order between Panels (A, B) illustrates genotype‐by‐environment interactions, reflecting species‐specific thermal niches and breeding strategies.

We applied the custom parameterized models to two hypothetical spring temperature regimes with mean diel temperatures of 16°C (cool) and 24°C (warm). Under the cool regime, wood frogs hatched earliest while green frogs lagged behind by up to 8 days—nearly double the developmental duration (Figure 4A). However, under the warm regime, predicted hatch dates clustered tightly, with all species hatching within 1 day of each other (Figure 4B). The effective value slopes (Figure 4C) reveal these underlying differences in developmental response curves, serving as species‐specific linearized reaction norms. Notably, the rank order of developmental timing in green frogs reverses across the two thermal regimes, a hallmark of strong G × E interaction.

This example demonstrates how **hatchR** can be used to explore evolutionary and ecological differences in thermal sensitivity across species. When combined with phylogenetic or trait‐based analyses, such reaction norms can reveal how developmental strategies align with species' ecological niches, helping to explain the persistence of closely related species across thermal gradients.

### 3.3 Local Adaptation: Cogradient Variation in Developmental Rates

As with interspecific comparisons, developmental models can be parameterized at the population level to examine local adaptation within species. In particular, **hatchR** allows researchers to compare how different populations respond to temperature, helping to reveal evolved differences in thermal sensitivity across environmental gradients.

**FIGURE 5:**
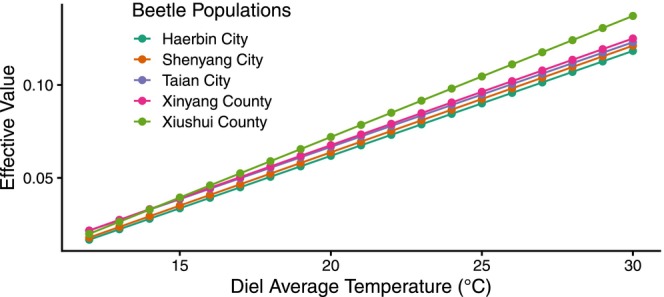
Effective value models for five populations of cabbage beetles (*Colaphellus bowringi*, females) along a latitudinal gradient in China (Tang et al. 2017). Models were parameterized from common garden experiments under constant temperatures. The southernmost population (Xiushui County) shows a consistently faster developmental rate than populations from more northerly sites, especially at warmer temperatures; a pattern is indicative of cogradient variation.

Here, we used data from a common garden experiment on cabbage beetles (*Colaphellus bowringi*, females) reared at six constant temperatures (16°C–28°C), spanning populations collected along a ~3440 km latitudinal gradient in China (Tang et al. 2017). Although all beetles were reared under identical conditions, developmental rates differed among populations, particularly at cooler temperatures. To visualize these patterns, we fit custom effective value models for five of the six populations (excluding Longnan, which duplicated the values from Xiushui). The resulting linearized effective value curves (Figure 5) show that beetles from Xiushui County (southernmost) had consistently faster development rates across temperatures than their more northerly counterparts, a pattern consistent with cogradient variation, where genetic and environmental effects on a phenotype act in the same direction (Conover et al. 2009; Sparks et al. 2022). These differences are especially pronounced at warmer temperatures, suggesting that local adaptation manifests differentially under certain environmental conditions. Moreover, the consistent rank order of developmental rates across the populations indicates heritable differences among populations which have and can continue to be selected upon in changing environments.

This example illustrates how **hatchR** can be used to explore the intersection of developmental plasticity and local adaptation in a novel framework. Such applications are useful for disentangling the genetic basis of thermal sensitivity, anticipating geographic responses to changing temperatures, and informing population‐specific management strategies.

## 4 Discussion

**hatchR** is a generalizable and flexible tool for predicting phenology across a broad set of poikilothermic organisms. The effective value framework accommodates nonlinear thermal sensitivity and allows researchers to estimate the timing of developmental milestones under real‐world, fluctuating temperature regimes with high accuracy and precision (Figure 1A,B). In this manuscript, we demonstrate how power‐law models can be fit using the fit_model() function, or defined externally and passed into the predict_phenology() function. We also illustrate how effective value models account for variable thermal environments and compensatory responses by organisms, which may create significant bias in commonly used thermal developmental models (Figure 1C,D). **hatchR** is distributed as an R package (R Core Team 2024; Sparks et al. 2025) for iterative and customizable processes, as well as a graphical user interface available via the **hatchR** Shiny app, expanding accessibility and encouraging broader adoption across disciplines.

We highlight the extensibility of **hatchR** through a compilation of parameterizable datasets spanning seven taxonomic classes and through three case studies that illustrate ecological, evolutionary, and conservation applications. These case studies span field data, phylogenetic comparisons, and experimental designs, demonstrating the tool's flexibility across data types and biological questions.

This modeling framework is largely designed to work within organisms' range of thermal optima. Specifically, the approach presented here ignores Jensen's inequality (Ruel et al. 1999), as models are parameterized with temperatures generally within thresholds suitable for their respective organisms. Organisms experiencing temperatures outside of those thresholds may respond in a manner that doesn't match the modeled relationship or adds additional error to the model (Colinet et al. 2015). However, the effective value framework is flexible with other modeling approaches, so reciprocation could be used with other non‐linear model forms fit outside of **hatchR** but then applied using the predict_phenology() function (see the **hatchR** manual for details; Sparks et al. 2025, 2026).

Other factors that may contribute to poor model performance include thermal damage from temperatures outside of thermal optima (Arnold et al. 2025) or the manner in which thermal variability is delivered to an organism (Steel et al. 2012; Kefford et al. 2022). As with any model of developmental timing, it is important to recognize that temperature is not the sole determinant of phenological events. For embryos, environmentally cued hatching is seen across taxa, wherein variation in hatch timing and developmental stage at hatching can result from myriad abiotic and biotic triggers apart from temperature (Sih et al. 2004; Warkentin 2011). While **hatchR** focuses specifically on temperature‐dependent development, it can be integrated with complementary datasets or experimental manipulations to investigate these more complex, context‐dependent interactions.

We attempted to verify model predictions for a wild population, but we could not find an example where we could match experimental data at an appropriate scale (e.g., for a locally adapted population) with recorded developmental data for that wild population. We believe the paucity of these data is driven by the difficulty in parameterizing and applying models for wild populations—a problem we believe **hatchR** is well positioned to address. The highly mechanistic relationship of these models—our hatch timing model for tailed frogs describes 99.6% of variance in hatch timing in our data. As an alternative, we used an example in which sockeye salmon were reared in a common garden experiment under thermal regimes matching their natal thermal environment. Models were developed with average temperatures over the course of development in these experiments and these were used to predict hatch timing using the “real‐world” thermal regimes (Figure 2). This example showed high concordance and low error, indicating these models should perform well once users can match appropriately scaled parameterizations with in situ empirical developmental data. The most highly variable regime was the one in which the model was least accurate, providing additional credence to the concern of how temperature is delivered to an organism. However, the difference was still small (8 days, or 6.8% of the developmental period), but worth additional investigation.

We encourage users to carefully consider how models are parameterized. As discussed in greater detail in documentation for Sparks et al. (2026), we recommend using experimental data with at least four distinct temperature treatments spanning a wide thermal range. This is critical for capturing the nonlinear shape of developmental rate curves and avoiding overfitting or extrapolation errors. Many studies we encountered—particularly older or gray literature—relied on only two temperature treatments (e.g., Qualls and Shine 1998; Kozák et al. 2009), which we do not recommend for model fitting due to the inability to detect curvature.

Given the wide potential applicability of **hatchR**, we especially emphasize its utility for rapidly developing species. For example, as shown in our phylogenetic case study, frog species with short incubation periods can be reared across multiple treatments in a single month, making them ideal for both research and teaching applications. Researchers can use these models to investigate thermal adaptation, phenological mismatch, or range‐wide trait variation. In educational settings, developmental models could be built for *Daphnia*, insects, or amphibians to introduce students to reaction norms, thermal performance, and evolutionary trade‐offs. Moreover, while **hatchR** currently focuses on pre‐feeding stages, the effective value approach could potentially be extended to post‐hatch phenological events—particularly when energy input is standardized (e.g., individuals reared under ad libitum conditions) (Lillehammer 1986).

## 5 Conclusion

**hatchR** is a broadly applicable tool for modeling temperature‐dependent development in poikilotherms. It addresses key limitations of traditional degree‐day approaches by accounting for non‐linear thermal response curves and enabling predictions under fluctuating thermal conditions. As global temperatures rise and the phenology of organisms shifts in response, tools like **hatchR** will be increasingly valuable for anticipating the ecological and evolutionary consequences of variable climates. While we provide numerous species‐level parameterizations and demonstrate a set of representative case studies, the full range of possible applications—across taxa, ecosystems, and educational settings—extends far beyond what we present here.

## Author Contributions

**Morgan M. Sparks:** conceptualization (equal), data curation (equal), formal analysis (equal), investigation (equal), methodology (equal), project administration (equal), resources (equal), software (equal), validation (equal), visualization (equal), writing – original draft (equal), writing – review and editing (equal). **Brian C. Leavell:** conceptualization (equal), data curation (equal), investigation (equal), writing – review and editing (equal). **Bryan M. Maitland:** conceptualization (equal), data curation (equal), formal analysis (equal), investigation (equal), methodology (equal), project administration (equal), resources (equal), software (equal), writing – review and editing (equal).

## Conflicts of Interest

The authors declare no conflicts of interest.

## Data Availability

**hatchR** is an open‐source software environment distributed via CRAN and additional details are hosted at the software website (https://bmait101.github.io/hatchR/). Data and scripts for this manuscript (Maitland and Sparks 2026) are available at Zenodo: http://doi.org/10.5281/zenodo.21682682. Site‐specific data for oviposition timing in Case Study 1 were not included at the request of the original authors of those data due to the sensitive nature of the species; however, they can be shared for individual requests.
